# Supplementary material for: Oncogene aberrations drive medulloblastoma progression, not initiation
Source: Nature. 2025 May 7;642(8069):1062–72. doi: 10.1038/s41586-025-08973-5 (PMC12222029; doi:10.1038/s41586-025-08973-5)
Supplement: Supplementary file 1 — Supplementary Tables 1–8. [file 41586_2025_8973_MOESM1_ESM.zip › 2024-01-00144D-s1/Supplementary Table Legends.docx]

**Supplementary Information**

**Supplementary Table 1.** Overview of target Group 3/4 medulloblastoma cohort with focus on snRNA-seq, snATAC-seq, scDNA/RNA and single cell spatial transcriptomics data.

**Supplementary Table 2.** Differentially expressed genes, specific for *MYC*, *MYCN* and *PRDM6* as well as non-*MYC/MYCN/PRDM6* subclones confirmed in minimum n=3 samples.

**Supplementary Table 3.** Cis-regulatory elements specific for *MYC*, *MYCN* and *PRDM6* as well as non-*MYC/MYCN/PRDM6* subclones confirmed in minimum n=3 samples. Annotation includes associated genes.

**Supplementary Table 4.** Summary of somatic mutation calling from singe nuclei RNA, ATAC and deep coverage ATAC data in n=3 MYC cases. Initially mutations were obtained from bulk WGS data.

**Supplementary Table 5.** Overview of target medulloblastoma ICGC cohort with focus on WGS data.

**Supplementary Table 6.** a) Genes associated to Group 3/4 medulloblastoma-specific chromosomal gains (gene active) and losses (gene inactive). b) Global differentially expressed genes specific for Group 3/4 tumors identified by contrasting gene expression on Group 3/4 -specific chromosomal gains and losses between tumor with and without the copy number change and between Group 3/4 medulloblastomas and other CNS tumors.

**Supplementary Table 7.** List of 100 target genes applied for the spatial single cell protocol.

**Supplementary Table 8.** Quality control overview of spatial single cell data.
