# Supplementary material for: Oncogene aberrations drive medulloblastoma progression, not initiation
Source: Nature. 2025 May 7;642(8069):1062–72. doi: 10.1038/s41586-025-08973-5 (PMC12222029; doi:10.1038/s41586-025-08973-5)
Supplement: Supplementary file 2 — Reporting Summary [file 41586_2025_8973_MOESM2_ESM.pdf]

nature portfolio

Corresponding author(s): Lena M. Kutscher
Stefan M. Pfister

Last updated by author(s): Feb 12, 2025

Reporting Summary

Nature Portfolio wishes to improve the reproducibility of the work that we publish. This form provides structure for consistency and transparency in reporting. For further information on Nature Portfolio policies, see our Editorial Policies and the Editorial Policy Checklist.

Statistics

For all statistical analyses, confirm that the following items are present in the figure legend, table legend, main text, or Methods section.

n/a	Confirmed	
☐	☒	The exact sample size (n) for each experimental group/condition, given as a discrete number and unit of measurement
☒	☐	A statement on whether measurements were taken from distinct samples or whether the same sample was measured repeatedly
☐	☒	The statistical test(s) used AND whether they are one- or two-sided<br>*Only common tests should be described solely by name; describe more complex techniques in the Methods section.*
☒	☐	A description of all covariates tested
☐	☒	A description of any assumptions or corrections, such as tests of normality and adjustment for multiple comparisons
☐	☒	A full description of the statistical parameters including central tendency (e.g. means) or other basic estimates (e.g. regression coefficient) AND variation (e.g. standard deviation) or associated estimates of uncertainty (e.g. confidence intervals)
☐	☒	For null hypothesis testing, the test statistic (e.g. F, t, r) with confidence intervals, effect sizes, degrees of freedom and P value noted<br>*Give P values as exact values whenever suitable.*
☒	☐	For Bayesian analysis, information on the choice of priors and Markov chain Monte Carlo settings
☒	☐	For hierarchical and complex designs, identification of the appropriate level for tests and full reporting of outcomes
☐	☒	Estimates of effect sizes (e.g. Cohen's d, Pearson's r), indicating how they were calculated

Our web collection on statistics for biologists contains articles on many of the points above.

Software and code

Policy information about availability of computer code

Data collection	Not applicable

Data analysis

Single nucleus RNA-sequencing and spatial data post analysis was performed in R 4.2 environment with the following packages: Seurat v4.1.3, dplyr v1.1.0, GSVA 1.46.0, Signac 1.10.0, InferCNV 1.10.1, scales_1.2.1 RImageJROI_0.1.2, data.table 1.14.6, stringr 1.5.0, Giotto v1.1.1., QuPath v0.3.2, ggplot2 3.4.0.

Whole genome sequencig data analysis was performed in R v4.2 with the following packages:
ggplot2 v3.5.1, bedr v1.0.7, openxslx v4.2.5, NBevolution v0.0.0.9000, ggpubr v0.4.0, ggsci v2.9, pammtools v0.5.8, tidyverse v1.3.2, mixtools v1.2.0, RColorBrewer v1.1.3, ggbeeswarm v0.6.0, survminer v0.4.9, survival v3.4.0, GSA v1.3.3, GSVA v1.44.5, msigdb v1.4.0, ExperimentHub v2.4.0, GSEABase v1.58.0, ComplexHeatmap v2.13.1, wesanderson v0.3.6, GenomicRanges v1.48.0, ggbio v1.44.1, MutationTimeR v1.0.2, VariantAnnotation v1.42.1, cdata v1.2.0, cowplot v1.1.1, mobster v1.0.0, CNAqc v1.0.0, HDInterval v0.2.2, moments v0.14.1,

Approximate Bayesian Computation was run in python v3.10.1 using pyABC v0.12.6

Cell border detection for Resolve Bioscience spatial images was performed with CellPose 2.0.5. Gene expression counts were extracted with custom Python scripts in python3.2 environment.

The source code materials for data analysis are shared via the repository:
github.com/kokonech/mbOncoAberrations

For manuscripts utilizing custom algorithms or software that are central to the research but not yet described in published literature, software must be made available to editors and reviewers. We strongly encourage code deposition in a community repository (e.g. GitHub). See the Nature Portfolio guidelines for submitting code & software for further information.

Data

Policy information about availability of data

All manuscripts must include a data availability statement. This statement should provide the following information, where applicable:

- Accession codes, unique identifiers, or web links for publicly available datasets
- A description of any restrictions on data availability
- For clinical datasets or third party data, please ensure that the statement adheres to our policy

The DNA whole genome sequencing mutation results were integrated from the corresponding medulloblastoma molecular landscape study deposited at European Genome-Phenome Archive under accession number EGAS00001001953. Single nuclei RNA and ATAC data available at GEO database under the accession numbers GSE253557 and GSE253573 accordingly. All raw images and processed data after cell segmentation from spatial transcriptomics experiments available at GEO database and can be accessed under the accession number GSE252090.

Research involving human participants, their data, or biological material

Policy information about studies with human participants or human data. See also policy information about sex, gender (identity/presentation), and sexual orientation and race, ethnicity and racism.

Reporting on sex and gender

Term sex is used for patients description, provided in annotation heatmap describing the target cohort. It is not used in the research.

Reporting on race, ethnicity, or other socially relevant groupings

The samples were collected from archives such as based on material availability, we do not have information on race or ethnicitiy for this cohort.

Population characteristics

The study focuses on medulloblastoma brain tumors, mean age : 5 years

Recruitment

Patients were included retrospectively based on the availability of fresh frozen tumour material.

Ethics oversight

This study was performed following the regulations of the ethics committee of the Medical Faculty of Heidelberg University.

Note that full information on the approval of the study protocol must also be provided in the manuscript.

Field-specific reporting

Please select the one below that is the best fit for your research. If you are not sure, read the appropriate sections before making your selection.

☒ Life sciences ☐ Behavioural & social sciences ☐ Ecological, evolutionary & environmental sciences

For a reference copy of the document with all sections, see nature.com/documents/nr-reporting-summary-flat.pdf

Life sciences study design

All studies must disclose on these points even when the disclosure is negative.

Sample size

n = 20

Sample size	Sample size is based on the availability of fresh frozen material of previously diagnosed MB G34 tumours with specific focus on cases with MYC,MYCN and SNCAIP/PRDM6 somatic alternations.
Data exclusions	No data was excluded in this study except of specific subsets not fitting quality control limits (e.g. low quality cells from single cell RNA/ATAC/spatial data)
Replication	For each target somatic change (MYC/MYCN/PRDM6) at least n=3 samples were included.
Randomization	Not applicable since the analyses were performed on collected available tumour material.
Blinding	All analyses were performed on retrospectively collected tumour material. No analyses involving living organisms were performed.

Reporting for specific materials, systems and methods

We require information from authors about some types of materials, experimental systems and methods used in many studies. Here, indicate whether each material, system or method listed is relevant to your study. If you are not sure if a list item applies to your research, read the appropriate section before selecting a response.

Materials & experimental systems

n/a	Involved in the study
☒	☐ Antibodies
☒	☐ Eukaryotic cell lines
☒	☐ Palaeontology and archaeology
☒	☐ Animals and other organisms
☒	☐ Clinical data
☒	☐ Dual use research of concern
☒	☐ Plants

Methods

n/a	Involved in the study
☒	☐ ChIP-seq
☒	☐ Flow cytometry
☒	☐ MRI-based neuroimaging

Plants

Seed stocks	n/a
Novel plant genotypes	n/a
Authentication	n/a
